# Unraveling the Role of Metals and Organic Acids in Bacterial Antimicrobial Resistance in the Food Chain

**DOI:** 10.3390/antibiotics12091474

**Published:** 2023-09-21

**Authors:** Andreia Rebelo, Agostinho Almeida, Luísa Peixe, Patrícia Antunes, Carla Novais

**Affiliations:** 1 UCIBIO—Applied Molecular Biosciences Unit, Laboratory of Microbiology, Department of Biological Sciences, Faculty of Pharmacy, University of Porto, 4050-313 Porto, Portugal; acr@ess.ipp.pt (A.R.); lpeixe@ff.up.pt (L.P.); 2 Associate Laboratory i4HB—Institute for Health and Bioeconomy, Faculty of Pharmacy, University of Porto, 4050-313 Porto, Portugal; 3 School of Medicine and Biomedical Sciences (ICBAS), University of Porto, 4050-313 Porto, Portugal; 4 ESS, Polytechnic of Porto, 4200-072 Porto, Portugal; 5 LAQV/REQUIMTE, Laboratory of Applied Chemistry, Faculty of Pharmacy, University of Porto, 4050-313 Porto, Portugal; aalmeida@ff.up.pt; 6 Faculty of Nutrition and Food Sciences (FCNAUP), University of Porto, 4150-180 Porto, Portugal

**Keywords:** copper, mercury, arsenic, organic acids, antibiotic resistance, food safety

## Abstract

Antimicrobial resistance (AMR) has a significant impact on human, animal, and environmental health, being spread in diverse settings. Antibiotic misuse and overuse in the food chain are widely recognized as primary drivers of antibiotic-resistant bacteria. However, other antimicrobials, such as metals and organic acids, commonly present in agri-food environments (e.g., in feed, biocides, or as long-term pollutants), may also contribute to this global public health problem, although this remains a debatable topic owing to limited data. This review aims to provide insights into the current role of metals (i.e., copper, arsenic, and mercury) and organic acids in the emergence and spread of AMR in the food chain. Based on a thorough literature review, this study adopts a unique integrative approach, analyzing in detail the known antimicrobial mechanisms of metals and organic acids, as well as the molecular adaptive tolerance strategies developed by diverse bacteria to overcome their action. Additionally, the interplay between the tolerance to metals or organic acids and AMR is explored, with particular focus on co-selection events. Through a comprehensive analysis, this review highlights potential silent drivers of AMR within the food chain and the need for further research at molecular and epidemiological levels across different food contexts worldwide.

## 1. Introduction

Antimicrobial resistance (AMR) is a critical global health challenge, ranked among the top ten public health threats worldwide [1]. This biological process occurs when microorganisms change over time (e.g., by acquisition of new genes or mutations) and no longer respond to antimicrobials. This makes infections more challenging to treat and increase the risk of disease spread, serious illness, and death [2]. Often referred to as the “silent pandemic” of the 21st century, the true global impact of AMR is difficult to assess, but estimates point to 700,000 deaths each year globally [3]. More recent estimates indicate that this number could be significantly higher, with 4.95 million human deaths associated with bacterial AMR in 2019, including 1.27 million directly linked to it [4], representing a much greater threat to public health than some infectious diseases such as malaria or HIV [4]. If no action plans are taken, projections indicate that the number of deaths due to AMR could rise to 10 million per year by 2050 [5]. In the European Union (EU) alone, bacterial AMR is estimated to be responsible for 33,000 deaths per year, with an economic impact of 1.5 billion/year in healthcare costs and productivity losses [6].

**Figure 1:**
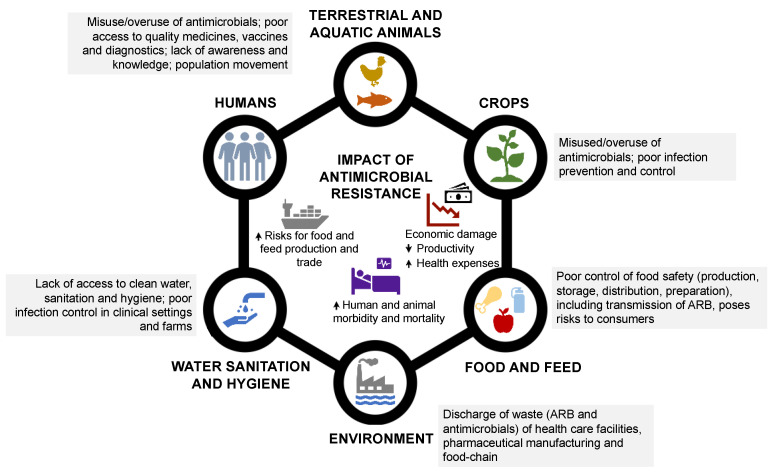
Drivers of antimicrobial resistance and their impacts at different levels: humans, terrestrial and aquatic animals, food and feed, crops, water sanitation and hygiene, and the environment (Adapted from [14]). Abbreviations: ARB—Antibiotic-resistant bacteria.

Since the discovery of antibiotics in the 1940s, the global threat of AMR has evolved dramatically over the past century [7]. While antibiotics are recognized as the greatest advance in the history of medicine [8], revolutionizing medical practice and saving millions of lives, their misuse and overuse, particularly in the medical, veterinary, and agricultural sectors, has triggered the emergence, escalation, and spread of AMR on a local and global scale (Figure 1) [9,10]. Such use creates selective pressure on bacteria, leading to the survival and proliferation of antibiotic-resistant bacteria (ARB), eliminating the susceptible ones, and promoting the exchange and spread of antibiotic resistance genes (ARGs) among multiple bacterial species through horizontal transfer events or bacteria vertical heritage [11].

Although the link between human or animal antimicrobial use and AMR seems clear- cut, this association is a complex process involving multiple events, including pathogen–drug and pathogen–host interactions, the high mutation rates of particular strains, the emergence and expansion of successful antimicrobial-resistant clones and/or mobile genetic elements (MGE), co-selection events by unrelated antimicrobials (e.g., different antibiotics or biocides), and the variable transmission rates of pathogens between humans, animals, and the environment [11]. Diverse pathogenic bacterial species, as well as the microbiota of humans, animals, and the environment, are active participants in these events and can act as important reservoirs and disseminators of ARGs in different settings [12,13].

In addition to antimicrobial misuse, other factors are also important drivers of AMR spread, including poor infection control practices (e.g., vaccination), hygiene or biosecurity measures in healthcare facilities or animal production settings, limited access to clean water and sanitation, environmental waste discharges, and poor food hygiene and safety practices (Figure 1). Also, the globalization of human, animal, and food products, as well as variable policies in different countries regarding antibiotic use and AMR surveillance in food production and other sectors, contribute to this threat [15,16,17]. All these events that facilitate the spread of AMR have significant multilayered implications, including for human and animal morbidity and mortality, the food and feed trade, and the economy in general (Figure 1).

Although these multiple drivers occur in different settings, the food production sector has long been recognized as one of the main environments for AMR expansion through ARB and MGE selection and evolution [18]. Such ARB can be introduced at any stage along the farm-to-fork continuum [19] and pose a potential risk to consumers. In fact, ARB from food and animals are important causes of human infections, highlighting the importance of global measures related to food hygiene and safety [20].

Changes in consumption trends due to rapid human population growth have led to the increase and globalization of the food supply [11], with the animal-food production industry accounting for approximately 70% of global antimicrobials sales worldwide [12,21]. Antimicrobials are used in different agri-food sectors and at different stages of production, both in intensive food-producing animals (terrestrial and aquatic) and crop productions [22]. However, while antimicrobials play a vital role in preserving animal health and welfare, as well as ensuring food safety and security, most of their use worldwide is to prevent rather than to treat infections (e.g., to compensate for poor farming practices) or, in specific countries, to promote animal growth [5]. Antibiotic use, including as veterinary agents, at subtherapeutic doses to increase the feed-to-weight ratio in animals or as pesticides in crop production, leads to the emergence/expansion of ARB in the food chain [13,18]. However, in addition to antibiotic use, AMR transmission routes are intricate and involve the participation of different players external to the food-producing animals, which can also promote the spread of AMR in the food chain. These include feed, workers, air/dust, equipment, water, soil, crops, rodents and other wildlife, and visitors, which can be vehicles or vectors of ARB into and out of farms or food processing plants. Ultimately, ARB can be transmitted to humans through contaminated food and water consumption, direct contact with animals, or exposure to water sources contaminated by agricultural and farm wastes [10].

Over time, numerous studies have identified ARB and/or ARGs of higher public health priority in food-production settings where animal or non-animal foods are produced or processed, including both pre-harvest (primary production) and post-harvest levels (such as slaughterhouses and processing plants) [13]. Among them, methicillin-resistant *Staphylococcus aureus* has been identified in livestock and poultry meat [23,24,25], vancomycin-resistant or linezolid-resistant *Enterococcus* in poultry and pork [26,27,28,29], *mcr*-1 colistin-resistant *Escherichia coli* or *Salmonella* in vegetables, unprocessed meat, livestock and farm environments [30,31,32,33], and carbapenem-resistant *Enterobacteriaceae* in vegetables and livestock [34,35], including *Salmonella enterica* serovar Typhimurium in pork [36,37], all bacteria that may pose serious risks to human and animal health. Despite growing concerns about the role of the food chain in the emergence and spread of AMR and the currently available surveillance data on animals, food, humans, and the environment, there is still limited information on the proportion of ARB strains or ARGs transferred and spread from the food chain to humans for most bacteria [38,39]. This knowledge gap makes it difficult to accurately assess the extent to which the food chain contributes to AMR transmission to humans [38]. Thus, effective AMR control requires a coordinated effort within and across countries to identify targeted interventions, improve surveillance and monitoring systems, raise stakeholder awareness, implement good practices to prevent and control AMR spread, use antibiotics responsibly, and strengthen governance [40].

Implementing restrictions on the use of antibiotics in food-producing animals is an important measure to curb the spread of AMR through the food chain, with numerous studies demonstrating a positive impact of limiting the use of antibiotics in reducing the prevalence of AMR in animal bacteria [41,42,43,44]. Global efforts and effective actions have been debated and implemented worldwide to address the issue of AMR in the food chain sector, with the EU taking a leading role in this commitment [6]. One of the main efforts is to reduce the use of antibiotics in food-producing animals by setting national reduction targets [e.g., the reduction of colistin in veterinary medicine to 5 mg/PCU (Population Correction unit) by 2021 in Portugal] [45]. Additional strategies include the restriction of antimicrobial drugs only for the treatment of certain human infections (e.g., carbapenems, glycopeptides, and oxazolidinones) [46], benchmarking antibiotic use at the farm level and promoting rational antibiotic stewardship, such as requiring susceptibility testing before use of some high-priority antibiotics [47]. Over the past few years, EU/EEA (European Economic Area) countries have made important progress in reducing the use of antibiotics in food-producing animals, resulting in a 47% decrease in sales between 2011 and 2021 [48]. This achievement is partly due to the actions taken in the early 1980s by some European countries such as Sweden, Norway, and Denmark, which were pioneers in restricting or banning the use of antibiotics as growth promoters in animal farms [49], leading to an EU-wide ban in 2006 through Regulation (EC) No. 1831/2003. More recently, a new milestone was reached with the interdiction of all forms of routine use of antibiotics in farm animals, including for prophylactic use [Regulation (EU) 2019/6 on veterinary medicinal products and Regulation (EU) 2019/4 on medicated feed]. With these actions, the EU aims to reduce by 50% the sale of antibiotics for farm animals and aquaculture by 2030 [50]. This paradigm creates new expectations regarding AMR reduction but also new challenges for the animal-farming sector to ensure animal safety, health, and welfare and at the same time to obtain the desired production level [51]. Apart from the reduction in antimicrobial use, other measures are essential to mitigate AMR, including the effective implementation of good hygiene practices and biosecurity measures [13,52]. Also, improving animal nutrition contributes to a good level of animal yield by reducing the vulnerability to bacterial infections and, consequently, the need for antimicrobials in animal husbandry practices [53].

In-feed supplementation with probiotics, enzymes, phytochemicals, antimicrobial peptides, metals and organic acids are among the available alternatives to antibiotics, with an important contribution to animal growth and disease prevention [54,55]. Some metals are essential nutrients for most animal species and are widely incorporated into animal feed to contribute to meeting nutritional requirements [56]. Among them, copper and zinc, are even added to feed in higher concentrations to act as growth promoters [56]. In addition to their use as feed additives, some metals have, for decades, been important antimicrobials in veterinary medicine, including arsenic (as coccidiostat) [57], copper (as fungicide and bactericide) [58], mercury (as preservative of veterinary drugs/bacteriostatic) [59], silver (as bacteriostatic/bactericide), and zinc (to treatment and prevention of diarrhea and skin infections) [60]. Currently metals, such as copper, continue to be promoted, including by official bodies [e.g., the European Medicines Agency (EMA) and the European Food Safety Authority (EFSA)], as alternatives to antibiotics due to their antimicrobial properties [47,61,62]. Thus, food production, as well as other anthropogenic activities, promote the release of metals into the environment, beyond their natural occurrence through biogeochemical processes [63,64,65]. The persistence of metals in the environment, due to their limited biodegradability, can lead to their accumulation in soil, water, and sediments, resulting in significant environmental contamination and selective pressure for ARB [56]. Therefore, new rules on metals use (e.g., zinc and copper) as feed additives or growth promoters have been implemented by the EU to control such events [66,67]. Other compounds, such as organic acids (e.g., peracetic acid) have been used as disinfectants for equipment and surfaces in food production environments [68,69,70], decontaminants on carcass surfaces following slaughter (e.g., lactic acid on bovines) [71], or feed additives (e.g., lactic and citric acids as preservatives) [72], with less environmental impact than other biocides [55].

**Figure 2:**
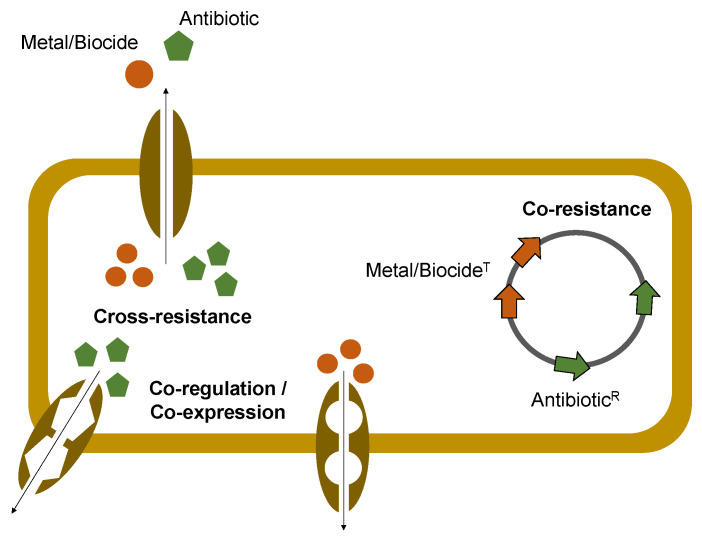
Mechanisms of metal/biocide and antibiotic co-selection: cross-resistance, co-resistance, and co-regulation/co-expression (Adapted from [75]). Abbreviations: R—Resistance, T—Tolerance.

Regardless of their importance in food production environments, metals or other compounds (e.g., biocides and organic acids) have been suggested to be associated with the co-selection and dissemination of ARB [73,74]. This association stems from the fact that many genes that confer tolerance to these chemical agents are frequently located in the same genetic elements as ARGs (co-resistance) (Figure 2). Also, other less frequently described co-selection mechanisms might be involved, including the occurrence of a single mechanism that may confer resistance to metals/biocides and antibiotics simultaneously (e.g., efflux pumps) (cross-resistance) or the presence of a common regulator responsible for controlling the expression of metal and antibiotic resistance systems (co-regulation/co-expression) (Figure 2) [75]. Additionally, exposure to low antimicrobial concentrations has been described to increase horizontal transfer events or the occurrence of bacteria genome mutations with an impact on AMR [73,76,77,78,79].

Despite the wide use or presence of metals and organics acids in the food chain, and of scattered literature showing their interplay with ARB, their role in the spread of AMR remains a debatable topic deserving a deeper analysis and reflection. Key research questions still needing clarification involve unequivocally identifying and separating homeostatic (tolerance to low concentrations) from acquired (tolerance to high concentrations) mobilizable genes that strongly contribute to bacterial adaptation and survival in environments with varying selective pressures of metals and organic acids. Additionally, it is important to clarify the variety of genetic contexts responsible for their successful spread among different bacterial taxa sharing the same ecosystems, as well as exploring the ecological factors favoring the co-occurrence and expression of metal or organic acid tolerance and ARGs. This review aims to provide a current and unique standpoint on the presence of copper and organic acids (e.g., widely applied in feed and biocides), as well as arsenic and mercury (long-term pollutants) in the food chain, their antimicrobial mechanisms and environmental factors enhancing their effects, and the variety of adaptive homeostatic and acquired tolerance mechanisms in diverse bacteria taxa occurring in the food chain. It presents detailed insights on metals and organic acids tolerance and ARGs’ interplay within diverse genetic contexts and bacterial taxa, with a focus on co-selection events. Through this comprehensive analysis, this review highlights potential silent drivers of AMR within the food chain context.

## 2. Metals

### 2.1. Copper

Copper (Cu) is an essential mineral for all living organisms [80], participating in various biological processes. In bacteria, it is found as a cofactor in proteins and enzymes due to its redox potential, acting as an electron donor/acceptor by alternating between the reduced cuprous form [Cu(I) or Cu^+^] and the oxidized cupric form [Cu(II) or Cu^2+^], critical for a wide range of metabolic and regulatory cellular functions [81,82,83] (e.g., electron transport, oxidative respiration, denitrification, etc.) [84,85]. However, in certain forms and concentrations, it can be toxic and inhibit or kill bacteria [86,87].

The antimicrobial properties of copper are well described [87], and its use dates back to ancient Egypt for the preservation of water and food, as well as for medical applications [88]. In the agri-food sector, copper-based compounds have been used as antimicrobials since the end of the 19th century, when its activity as a fungicide was first described when applied within the “Bordeaux mixture” in vineyards [89]. Since then, it has been widely used in pesticides and fertilizers [90,91]. Although the role of copper as an antimicrobial agent was widely recognized in the past, it lost significance with the advent of antibiotics [92]. However, the biocidal properties of copper against a wide range of pathogens have made it regain importance as a promising alternative in the fight against the spread of multidrug-resistant (MDR) bacteria [92]. Among the currently authorized copper applications in the EU are several copper-based biocidal products not intended for direct application to humans or animals [93]. In recent years, the use of copper plating of surfaces, including in the food and medical sectors [94,95,96,97], has been proposed as a more effective measure to limit bacterial adhesion than stainless steel [87], being the first solid antimicrobial material registered with the U.S. Environmental Protection Agency [92]. Other antimicrobial applications of copper have been made, most in clinical settings (e.g., medical devices such as copper-impregnated fabrics) [98,99,100,101].

Although copper is commonly known for its antimicrobial properties, it also plays a crucial role in human and veterinary medicine in the treatment of nutritional deficiencies [58]. In food-producing animals, feed is routinely supplemented with copper not only to meet the animals’ nutritional needs but also to improve their growth performance by modulating the gastrointestinal tract microbiota, leading to improved nutrient absorption [102]. Varying concentrations of copper are used, depending on the species, age group, and feed composition, as copper can interact with other nutrients, including other metals (e.g., zinc, iron, calcium, and molybdenum) and phytates [103]. As an example, the maximum concentration allowed in poultry feed is 25 mg Cu/kg, while in piglets up to 4 weeks after weaning, it is 150 mg Cu/kg, and from the fifth to the eighth week after weaning, it is 100 mg Cu/kg [66]. Traditionally, feed supplementation with inorganic trace mineral (ITM) copper has been used as a cost-effective solution [104,105], but the use of other forms, mainly organic species (organic trace mineral—OTM) and copper nanoparticles, has been increasing, as they present higher bioavailability, improving animals’ growth performance, with a lower environmental impact [105,106,107]. The application of copper nanoparticles has also been exploited in the food industry and agriculture sectors, mainly to prevent microorganism spoilage (e.g., in food packaging) [108] and as agro-nanochemicals (e.g., fertilizers and pesticides) with a larger specific surface area than conventional forms [109]. However, the widespread use of copper-based compounds in many anthropogenic activities has led to copper’s accumulation in different ecosystems, making it a pollutant and potentially toxic to many organisms, including bacteria.

Copper poses a unique challenge to bacteria due to its dual nature—it is an essential trace mineral, but it can also be cytotoxic when present in excess. This ambivalence highlights the importance of the strict regulation of cellular copper levels [110]. Maintaining copper homeostasis requires a delicate balance between providing the required dose of the micronutrient while avoiding toxic excess [56,111]. Although the mechanisms of how copper ions affect bacteria are still not fully understood, it seems that the cycling between the cupric [Cu(II)] and the cuprous [Cu(I)] states can disturb the intracellular redox potential, the main cause of cytotoxicity. In particular, the intracellular soluble fraction of copper [Cu(I)], via a Fenton-like reaction, catalyzes the formation of superoxide (O_2_^−^) and other reactive oxygen species [hydroxyl radicals (OH·) and hydrogen peroxide (H_2_O_2_)], which are responsible for lipid peroxidation, protein oxidation, and DNA damage [112]. Under low oxygen conditions, the reduced ionic species Cu(I) is prevalent and is highly toxic, showing a great affinity for thiolates and other sulfur-containing compounds, disrupting the binding of iron–sulfur (Fe-S) clusters, leading to poor protein metallation, protein inactivation, and ultimately to dysfunctional cell metabolism [112,113,114]. In human macrophages, copper is pumped to their phagosomes after engulfing pathogenic bacteria to induce bacteria death by oxidative stress [115].

Copper can often enter bacterial cells in an unspecific manner by using other metal uptake systems, making it difficult for bacteria to limit the amount of copper entering the cytoplasm [56]. Bacteria have evolved several mechanisms implicated in the uptake, internal traffic, storage, and efflux of copper from the cell, including the extracellular sequestration of copper ions, the relative impermeability of the outer and inner bacterial membranes to copper ions, the presence of metallothionein-like copper-scavenging proteins in the cytoplasm and periplasm, and the active extrusion of copper from the cell [92].

The extrusion of excess cytoplasmic copper by homeostatic mechanisms appears to be the main defense mechanism in bacteria, a process that has been extensively studied in both Gram-positive and Gram-negative bacteria [92]. Specifically, copper efflux occurs through transporters, members of the P_1B-1_-ATPase subfamily [Cu(I) transporters] of P_1B_-ATPases [116]. The first copper-transporting ATPases were described in *Enterococcus hirae* [117,118], represented by the cop operon (*copYZAB*), which is formed by four genes coding for the following proteins: CopA and CopB, responsible for the uptake and removal of excess Cu(I) from the cytoplasm, respectively [119]; CopZ, a chaperone responsible for intracellular copper transport; and CopY, a promoter regulator [120,121]. Unlike Gram-positive bacteria, which lack a periplasmic space and an outer membrane, Gram-negative bacteria require additional mechanisms to deal with the presence of copper in the periplasm. In the most studied Gram-negative bacterium, *E. coli*, in addition to the presence of the Cu(I)-translocating P-type ATPase CopA in the cytoplasmic membrane, responsible for pumping excess Cu(I) from the cytoplasm to the periplasm [122], there is also the CusCBA multicomponent copper efflux system and CueO multicopper oxidase. These two systems are chromosomally encoded and play important roles in controlling the copper level and redox state, respectively [56]. Since CueO acts only in the presence of oxygen, presumably oxidizing Cu(I) into the less toxic Cu(II) [56], the CusCBA transport complex is important to copper detoxification from the periplasm in the absence of CueO [123]. In *Salmonella*, copper defense determinants are quite similar to those of wildtype *E. coli*, also containing CopA and CueO. However, most *Salmonella* strains do not contain the CusCBA system, instead having the periplasmic copper-binding protein CueP [112].

**Figure 3:**
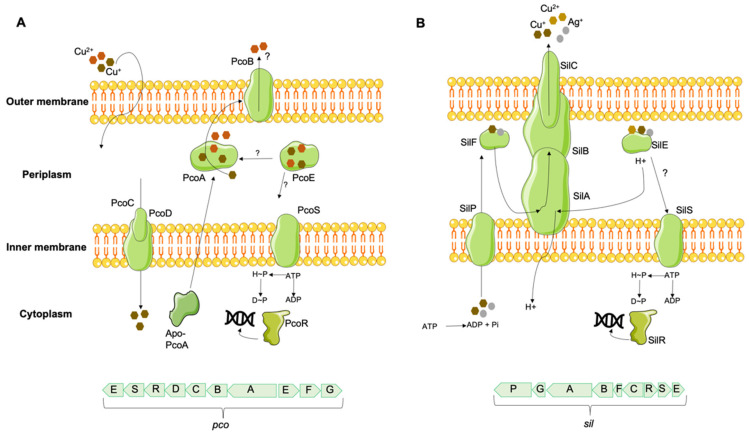
Mechanisms of copper tolerance associated with the *pco* (**A**) and *sil* (**B**) genes clusters. The genes and their transcriptional and translation directions are indicated below the illustration. Genes with unknown functions are not represented (Adapted with permission from [130]). (License number 5627591056231 attributed by Springer Nature) The figure was partly generated using Servier Medical Art, provided by Servier, licensed under a Creative Commons Attribution 3.0 unported license.

In environments with high copper concentrations, which would overwhelm chromosomally encoded copper metabolic systems, some bacteria have acquired copper tolerance mechanisms, regulated mainly by extrachromosomal loci [124]. The first mechanism described in Gram-negative bacteria was identified in the pRJ1004 plasmid of an Australian pig *E. coli* isolate [125], linked to the presence of the *pco* (plasmid-borne copper resistance) system. This system includes different structural proteins, including PcoA, a periplasmic multicopper oxidase, PcoB and PcoD, outer and inner membrane proteins, respectively, and PcoC and PcoE, two periplasmic proteins [125,126,127,128]. While PcoE is responsible for temporarily sequestering excess copper [128], PcoC is also capable of transferring it to the membrane-bound PcoD [56]. In turn, PcoD catalyzes the uptake of Cu(I) into the cell, which is incorporated into PcoA and exported to the periplasm, where it is detoxified either by sequestration or oxidation and removed via PcoB (Figure 3) [129]. A two-component regulatory system, PcoRS, seems to be responsible for the transcription of PcoABCD proteins [126], while the chromosomally encoded CusRS system regulates the transcription of the PcoE protein [128]. Two additional proteins, PcoF and PcoG, corresponding to a putative copper-binding protein and a putative metallopeptidase, respectively, may be present, but their role has yet to be determined [130]. The *pco* gene cluster encodes proteins responsible for periplasmic copper management, being dependent on the supply of copper by the cytoplasmic CopA protein to confer copper tolerance to bacteria [110]. Contiguous to the *pco* system in pRJ1004 is the *sil* gene, first described in the *S*. Typhimurium plasmid pMG101, and initially linked to silver tolerance [131]. The Sil system includes a SilCBA efflux complex responsible for exporting Cu(I) and Cu(II) from the periplasm, three periplasmic proteins, SilE [homolog to PcoE, presumably to bind Cu(I) and Cu(II)], SilF, and SilG, the first two acting as chaperones of the SilCBA complex and the last one with an unknown function, as well as a P-type ATPase SilP that transports copper and silver ions from the cytoplasm to the periplasm [132]. The two-component membrane sensor and transcriptional responder SilRS appear to be involved in silCFBAGP expression [130]. The occurrence of *sil* efflux systems is associated with a CuSO_4_ tolerance phenotype in several *Enterobacteriaceae* under anaerobic conditions, where the more toxic form Cu(I) is predominant, a distinct feature of isolates carrying *sil* ± *pco* genes in comparison with those without it [44,133,134,135]. A minimum inhibitory concentration (MIC) for CuSO_4_ between 16 and 36 mM has been described in isolates with *sil* ± *pco*, contrasted with a MIC_CuSO4_ between 2 and 12 mM in isolates without these genes [44,133,134], with a proposed CuSO_4_ tolerance cutoff ≥16 mM to differentiate isolates with and without *sil* ± *pco* gene clusters, under anaerobiosis [44,134].

Since the entire *sil* determinant confers copper tolerance, the contiguous 20-gene clusters of *pco*+*sil* have been referred to as copper-pathogenicity islands [130]. Although the *pco*+*sil* determinants were initially identified in plasmids, it is worth noting that this gene cluster may also be located on the chromosome [133,134], due to the bacteria’s genetic plasticity, which is often facilitated by the presence of Tn7-like transposons [134,136,137]. Several studies have been describing the wide occurrence and distribution of *sil*-*pco* clusters in diverse species and multiple environments, including food and food-producing animals [134,138], hospitals and urban wastewater [139], fresh water [140], veterinary clinical settings [141], and clinical human samples [134].

**Figure 4:**
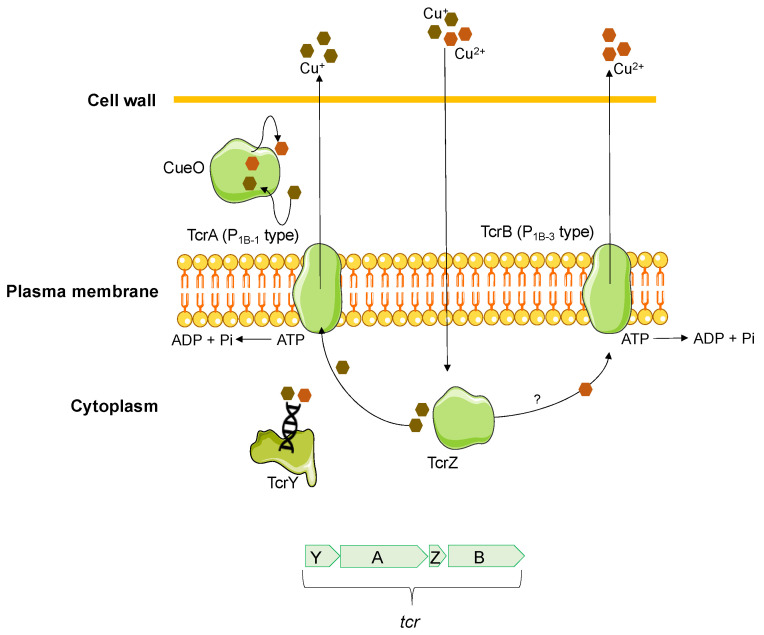
Representation of genes and protein products associated with the *tcrYAZB* operon and CueO multicopper oxidase protein in *Enterococcus* spp. The *tcrYAZB* operon genes and their transcriptional and translation directions are indicated below the illustration (Adapted from [129]). The figure was partly generated using Servier Medical Art, provided by Servier, licensed under a Creative Commons Attribution 3.0 unported license.

Gram-positive bacteria with high acquired tolerance to copper have also been described, namely in several species of *Enterococcus* genus. The most characterized gene is the plasmid-encoded *tcrB* (transferable copper resistance gene B) initially identified in an *E. faecium* isolate from pigs in Denmark [142]. The *tcrB* gene codes for an efflux pump, presumably belonging to the P_1B-3_-ATPase subfamily of copper transporters P_1B_-ATPases, which is activated mainly by Cu(II) and to a lesser extent by Cu(I) [129,143]. This gene is part of the *tcrYAZB* operon (homologous to the *copYZAB* copper-homeostasis gene cluster of *E. hirae*) [144], together with the *tcrA* gene, an additional P_1B_-ATPase of the P_1B-1_-ATPase subfamily and responsible for Cu(I) export, the *tcrZ* gene, which encodes a cytoplasmic copper chaperone (TcrZ) responsible for Cu(I) transport, and the *tcrY* gene, a copper-dependent regulator (TcrY) involved in controlling operon expression (Figure 4) [142,144]. These copper-tolerant determinants are often flanked by insertion sequences, allowing their transferability [145,146,147].

As the *sil* efflux systems, the acquisition of the *tcrYAZB* operon represents a clear advantage for bacteria in anaerobic environments, allowing them to survive in higher Cu concentrations [148]. *Enterococcus* spp. carrying *tcrYAZB* operon have shown an MIC_CuSO4_ between 16 and 36 mM, while in isolates without these genes, the MIC_CuSO4_ ranged between 4 and 12 mM [148,149,150]. Thus, a CuSO_4_ tolerance cutoff ≥16 mM was proposed to differentiate isolates with and without the *tcrB* gene, under anaerobic conditions [148]. In the vicinity of the *tcrYAZB* operon is often a multicopper oxidase (CueO), potentially involved in the oxidation of Cu(I) to Cu(II) [145].

As in Gram-negative bacteria, the *tcrYAZB* operon genes are located mainly in plasmids [142,146,149], unlike chromosomal genes related to copper homeostasis [151]. Since the first description of the *tcrYAZB* operon in the pA17sv1 plasmid of an *E. faecium* from a healthy pig [144], the presence of the *tcrB* gene has been mainly associated with *Enterococcus* genus isolates from food-animal production environments [145,146] and foodstuffs [145,149,152], with few studies describing its occurrence in humans (clinical and community isolates) and aquatic environments [145,148].

A major issue is that copper tolerance has been strongly associated with ARB in different environments (e.g., aquatic, animal-food production, agri-food, and clinical settings) [153,154,155]. Co-selection of copper tolerance genes and ARGs often occurs because they all share the same genetic elements [146,150,156]. Shortly after the first description of the *tcrB* gene, a link to macrolide and glycopeptide resistance was established by the co-occurrence of such resistance determinants on the same conjugative plasmid of porcine *E. faecium* [142,156]. More recently, other ARGs [e.g., *vanA*- vancomycin; *tet(M)* or *tet(L)*-tetracycline; *aadE*-streptomycin; *aac(6′)-Ie-aph(2″)-Ia*-gentamycin] have also been described in the same *Enterococcus* plasmids as the *tcrYAZB* operon and other metals in *Enterococcus* spp. of the food chain and other niches [149,150]. A single description of *tcrYAZB* on the chromosome is available for *E. faecalis* from poultry meat alongside mercury (*merA*) tolerance genes [149]. Plasmids carrying *sil* ± *pco* genes (and other metal tolerance genes, including to mercury—*mer* genes) and ARGs for beta-lactams (*bla*_TEM-1_, *bla*_CTX-M_), aminoglycosides [*aac(3)*, *aadA*], sulfonamides (*sul*), trimethoprim (*dfrA*), chloramphenicol (*cmlA*), and tetracyclines (*tet*) have also been described in *E. coli*, *Klebsiella pneumoniae,* and *Salmonella* isolates from food-production environments and human sources [133,134,157,158]. In addition, the chromosomal co-localization of copper (*pco*+*sil*) with other metal tolerance genes (e.g., *mer*) and ARGs for beta-lactams (*bla*_TEM-1_), aminoglycosides (*aadA*, *str*) sulfonamides (*sul*), trimethoprim (*dfrA*), and tetracyclines (*tet*) was described in *Salmonella* isolates from various sources (animal-food production; foodstuffs; humans) [133,134]. Cross-resistance and co-regulation mechanisms have been poorly described, with some studies suggesting the role of efflux systems (e.g., membrane transporters belonging to the RND family) in the extrusion of both copper and antibiotics (e.g., cefotaxime) in some Gammaproteobacteria [159,160] and overexpression of some binding proteins (e.g., Rob encoded by *robA* gene) associated with increased resistance to metals (including copper) and multiple antibiotics (e.g., tetracycline, chloramphenicol) in *E. coli* [161].

### 2.2. Arsenic

Arsenic (As) is a metalloid naturally present in the earth’s crust and widely distributed in soil, sediments, water, air, and living organisms [162,163]. Unlike other elements (e.g., copper and zinc), arsenic is not required for biological functions in most bacteria, exerting a toxic effect on the cell [164,165]. The toxicity of arsenic greatly depends on its oxidation state, and it can occur in four valence states: As^3−^ (arsine gas, AsH_3_), As^0^ (elemental arsenic), As^3+^ (trivalent arsenic or arsenite), and As^5+^ (pentavalent arsenic or arsenate) [166]. Arsenite and arsenate are the predominant species under reduced and oxygenated conditions, respectively, the former being 100 times more toxic than the pentavalent form [166].

Regardless of its ubiquitous distribution and the contribution of natural processes to increasing environmental arsenic contamination (e.g., mineralized and mined areas, volcanogenic activity, thermal springs, and Holocene alluvial sediments) [167], it is human activity that has greatly contributed to increase the arsenic concentrations in different environments [163]. Arsenic or arsenic-based compounds have historically been used in a range of applications, including pharmaceuticals, wood preservatives, agricultural chemicals (e.g., pesticides, cotton desiccants, defoliants, and soil sterilants) and in industry (e.g., mining and metallurgy) [162]. Inorganic arsenic compounds have been used in medicine since 2000 BCE, when arsenic trioxide (As_2_O_3_, commonly referred to as ATO) was used as both a drug and a poison [168]. Over time, the use and development of arsenic in medicine has evolved, with important milestones including its use by Hippocrates to treat skin cancers (using orpiment—As_2_S_3_, and realgar—As_4_S_4_) and its recommendation by Paracelsus for use in medicine [168]. After the 17th century, ATO became widely used as a drug to cure headaches and specifically to treat trypanosomiasis, syphilis, and leukemia [168]. Currently, ATO is still used as an anticancer chemotherapeutic agent for hematological diseases, listed as one of the essential medicines by the World Health Organization [169]. Although arsenic has this history of use in medicine, it is the agricultural and industrial sectors that have contributed the most to arsenic environmental pollution. In agriculture and animal farming, arsenic-based compounds have been extensively used in pesticides [e.g., sodium arsenite or sodium arsenate, Na_2_HAsO_3_/Na_2_HAsO_4_; calcium arsenite or calcium arsenate, Ca(AsO_2_)_2_/Ca_3_(AsO_4_)_2_], as coccidiostats, and as a feed additive, mainly in the poultry and swine industries [57,168,170]. Roxarsone, a pentavalent nitroaromatic arsenical, has been used exclusively for animal husbandry, particularly poultry, to promote growth, treat coccidiosis, and prevent gastrointestinal infections [57]. Despite possible accumulation in animal meat [57], most of the roxarsone ingested by animals is excreted in feces and urine, which might contribute to its accumulation in and around the animal production environment (e.g., manure, waste lagoons, and amended soils) [171,172]. For this reason, roxarsone is now banned in several countries around the world (e.g., EU countries, the USA, and China) [173,174].

Although many arsenic compounds are no longer used, their residues persist from past activities. A recent study showed that arsenic concentrations in more than half of European agricultural soils exceeded the threshold of 5 mg/kg [175], posing a threat to the environment, food safety, and human health. Moreover, concentrations found in animal-production environments (e.g., total arsenic in manure: ~0.016–2.5 mM; sludge: ~0.15 mM; feed: ~0.0003–0.174 mM) [176,177,178,179], suggest that arsenic may create selective pressure on bacteria in these environments, favoring the selection of those with tolerance to arsenic (and other metals), with particular concern for MDR zoonotic bacteria [180].

**Figure 5:**
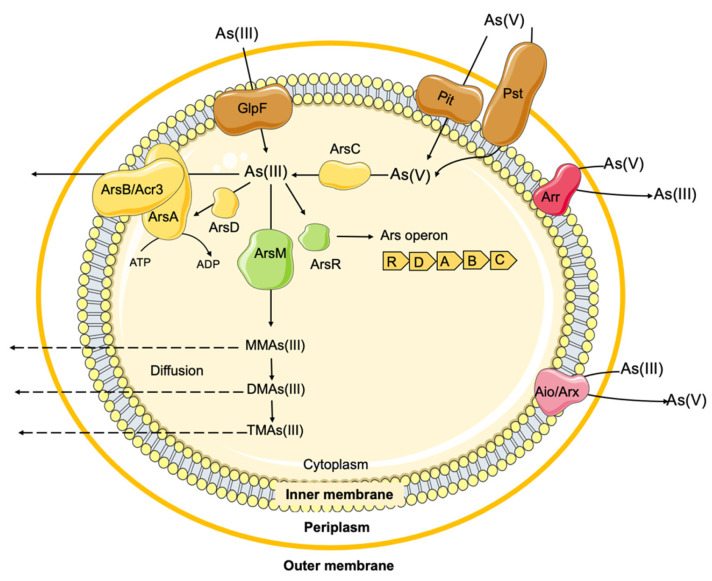
Arsenic detoxification and respiratory metabolic pathways in bacteria. Four different systems might be involved in arsenic biotransformation pathways. Inorganic and organic arsenic detoxification pathways include the arsenic resistance efflux system (*ars* operon) (yellow) and arsenic methylation (green), respectively. The respiratory oxidization of As(III) to As(V) and the reduction of As(V) to As(III) are represented by the Aio/Arx (light pink) and Arr systems (dark pink), respectively. Uptake of As(V) and As(III) is represented by the phosphate transporters (Pit or Pst) and by the aqua-glycerolporins (GlpF) (brown) (Adapted with permission from [183]). Abbreviations: MMAs(III), monomethylarsonous acid; DMAs(III), dimethylarsinous acid; TMAs(III), trimethylarsine. (License number 5627600311910 attributed by Springer Nature). The figure was partly generated using Servier Medical Art, provided by Servier, licensed under a Creative Commons Attribution 3.0 unported license.

Throughout Earth’s evolutionary history, bacteria have always been exposed to arsenic in different environments and have evolved numerous mechanisms to deal with it, either through detoxification or metabolic pathways [181,182]. Several arsenic biotransformation systems have been identified in bacteria, most of which are associated with detoxification processes. These include the arsenic resistance efflux system (*ars*), arsenic methylation and associated pathways (e.g., *arsM*), as well as metabolic processes such as arsenite oxidation (aio/arx) and reduction (arr) systems (Figure 5) [181].

Arsenic metabolic pathways involving biotransformation between As^3+^ and As^5+^ (Aio/Arx and Arr systems), represent an important energy-generating process in the respiratory process of some bacteria [182,184]. However, for most bacteria, arsenic is not essential, which explains the absence of specific arsenic uptake systems [165]. In fact, the analogy of some arsenic species with other molecules allows arsenic entrance into bacterial cells via nonspecific intrinsic transporters [185]. For example, arsenate is a phosphate analogue, entering into cells through phosphate transporters (Pit or Pst) (Figure 5) and inhibiting phosphorylation reactions (such as glycolysis and ATP production) [186]. However, it is unstable and can rapidly dissociate into the more toxic trivalent arsenite (As^3+^) [187]. Arsenite has a structural similarity to glycerol and enters the cell via aqua-glycerolporins (GlpF), the glycerol transport system (Figure 5) [165,181]. The greater toxicity of arsenite is related to its ability to bind strongly with sulfhydryl groups in proteins, impairing the function of many proteins important for biochemical processes, and binding weakly to other small thiol molecules (glutathione, lipoic acid, and cysteine), affecting respiration [184,186].

To cope with the continued exposure to arsenic toxicity, most bacteria have evolved and acquired genes for arsenic detoxification, mostly encoded by *ars* operons (Figure 5), often found among prokaryotic genomes, either on chromosomes or on plasmids of Gram-positive and Gram-negative bacteria [165,181,184,188], which reflects its ubiquitous presence in nature. The first description of arsenic tolerance genes occurred more than 50 years ago when a clinical strain of *S. aureus* was identified as carrying a plasmid (pI258) conferring tolerance to arsenate, arsenite, and other metals and resistance to antibiotics [189]. Shortly thereafter, another plasmid (R773) identified in a clinical strain of *E. coli* also revealed the occurrence of arsenic tolerance genes [190]. In both cases, *ars* operons involved in the arsenic tolerance phenotype were identified, encoding homologous proteins, but with different configurations: the three-gene *arsRBC* operon in the *Staphylococcus* pI258 plasmid and the extended five-gene *arsRDABC* operon in the *E. coli* R773 plasmid [184]. In fact, several genomic configurations of *ars* operons have been described and suggested to be strain-specific [165,184]. Most *ars* operons are involved in inorganic arsenic detoxification, although coupling with other *ars*-related genes also allows for organoarsenicals’ detoxification (Figure 5) [181]. In both types of *ars* operons, the core genes include a trans-acting transcriptional repressor protein (ArsR) that binds to the promoter region of the *ars* operons, an arsenite efflux pump (ArsB) and an arsenate reductase (ArsC) (Figure 5) [184]. ArsR interacts with arsenite, dissociating the repressor protein from DNA, thereby downregulating the transcription of other *ars* operon genes [184,191]. ArsB is an integral membrane protein responsible for the extrusion of arsenite [As(OH)_3_/H^+^ antiporter) from the cell cytoplasm, representing the basic mechanism of arsenite detoxification by decreasing its accumulation [192]. ArsB activity can involve two types of energy sources: acting independently on the arsenite transport channel, using the membrane potential to catalyze the extrusion of As^3+^ from the cell; or acting in conjugation with ArsA (in the case of operons *arsRDABC*), to potentiate arsenic tolerance to a higher degree [181]. Specifically, the ArsA ATPase protein catalyzes the hydrolysis of ATP, which energizes the arsenite efflux pump, forming the ArsA-ArsB membrane-bound complex (Figure 5). The ArsC protein is an arsenate reductase enzyme, capable of reducing intracellular arsenate to arsenite, which will then be extruded out of the cell through the ArsB pump [193]. Finally, the ArsD protein, which occurs in the extended *ars* operons (*arsRDABC*), is a metallochaperone responsible for sequestering cytosolic arsenite and transferring it to the ArsA subunit of the efflux pump, increasing the efficiency of arsenic extrusion (Figure 5) [192].

Genomic analysis has helped to identify the existence of atypical *ars* clusters [194,195] or the occurrence of additional genes associated with these clusters and involved in arsenic tolerance genes, including the *acr3* gene [196,197]. Acr3 (also known as ACR3 or ArsY) is a member of the BART (bile/arsenite/riboflavin transporter) superfamily, first reported in the *arsRBC* operon of *B. subtilis* as a typical ArsB membrane protein (Figure 5) [184]. In fact, the literature often describes members of the Acr3 family as ArsB-type, even though they do not exhibit significant sequence similarity to ArsB [198]. While the ArsB-type is mostly restricted to bacteria, including Bacillota (formerly Firmicutes) and Pseudomonadota (formerly Proteobacteria) [180,199,200], the Acr3-type family has a wide distribution, also found in archaea and eukaryotes (mainly fungi and some plants) [187,201,202]. Interestingly, a predominance of *acr3* over *arsB* genes was found in arsenic-tolerant bacterial isolates from arsenic-contaminated soils and, in some cases, concurrently with the *arsB* gene [203]. However, no evidence of the coexistence of the two transporters encoded in the same operon has been reported so far [202]. As with the ArsB-type, Acr3 can also couple with ArsA to form a more efficient arsenite efflux system [201]. A phenotype of increased arsenate (sodium arsenate Na_2_AsO_4_) tolerance was observed in Gram-positive (*Enterococcus* spp.) and Gram-negative (*Salmonella*) bacteria with arsenic tolerance genes (*arsA*, *arsB* or *acr3*) compared to those without these genes, with MICs ranging between 8 and ≥128 mM and between 0.5 and 4 mM, respectively, regardless of the atmosphere used (aerobic or anaerobic) [150,180].

The wide distribution of arsenic tolerance genes in bacteria from diverse sources (environment, food, and clinical) reflects not only the ubiquitous nature of this metal but also the bacteria’s adaptive characteristics. Arsenic tolerance genes (*arsA*/*arsB*/*acr3*) have been predominantly found in bacteria from natural environmental sites, regardless of whether they had a history of arsenic contamination, including soils (from forests or close to gold mining activities or geothermal effluents), creek water, and sewage [200,203,204,205]. Additionally, other contexts have been associated with the occurrence of arsenic tolerance genes, such as clinical (e.g., human samples and clinical settings) [141,206] and food-associated environments (e.g., food-producing animals, processing plants, and food products) [206,207]. In animal-food production environments, arsenic can accumulate and persist in sublethal concentrations, leading to long-term selective pressure on bacteria, which favors those with reduced susceptibility to arsenic and other antimicrobials (metals and antibiotics) [154]. In fact, there is growing evidence of the wide dispersion of arsenic tolerance genes in these environments, ranging from animals to other variable stages in food production, including raw, processed, and ready-to-eat animal products (e.g., swine, poultry, and cattle), associated and not associated with foodborne pathogens [207,208,209].

The co-localization of arsenic and other metal tolerance operons (e.g., mercury and copper) in the same genetic context have been described, either in plasmids or in chromosomal regions. These genetic regions have been pointed out as potential hotspots for the accretion of metal tolerance genes, either in bacteria with an environmental lifestyle (e.g., *Alteromonas* sp.) or food-chain associated bacteria (e.g., *Listeria* sp., *Salmonella* sp.) [206,210]. Furthermore, arsenic tolerance genes have been described as being on the same MGE as other metal tolerance genes or ARGs, including in plasmids (e.g., *E. coli*, *Klebsiella*, *Listeria monocytogenes*, *E. faecalis*) [44,188,211], or ICEs (Integrative Conjugative Elements) (e.g., *S*. Typhimurium) [212]. The variability of MGE-carrying arsenic tolerance genes may favor their horizontal transfer between bacterial hosts. Also, when integrated and fixed in the chromosome, arsenic tolerance genes can confer a lower fitness cost to bacteria and be spread by vertical transmission. In all cases, there is a selective advantage for bacterial survival, particularly in food-animal production or other metal-polluted environments. In fact, arsenic-polluted environments (e.g., water reservoirs and urban soils) have been described as contributing to the co-selection of ARGs [e.g., for aminoglycosides—*aadA*/*aacC*, beta-lactams—*bla*_CMY_/*ampC*, MLSB—*erm(F)* tetracyclines—*tet(B)*] and of mobilizable or MGE (e.g., integron—*intI-1*, transposon—Tn*21*/Tn*22*/Tn*24*/Tn*614*) [213,214]. The occurrence of arsenic and other metals (e.g., copper, zinc, cadmium, lead) in a Chinese poultry production environment has also recently been found to have a greater impact on metal tolerance genes and ARGs gene composition than some antibiotics, showing a positive correlation between arsenic concentrations and resistance genes to aminoglycosides [*aac(6′)-Ia*], macrolides (*erm35*), bacitracin (*bacA*) and, in particular, with resistance genes to tetracycline (*tet* genes), probably promoted by co-selection events [154].

### 2.3. Mercury

Mercury (Hg) is a highly toxic heavy metal widely dispersed in nature [215]. Like arsenic and other heavy metals, mercury is a non-essential element for living organisms, with no known beneficial function for cells [216]. The toxicological properties of mercury depend on the different chemical forms in which it can occur [217]. In the environment and in biological systems, mercury can be present in three oxidation states, namely, elemental mercury (Hg^0^) (known as metallic mercury, a highly volatile liquid at room temperature), and the mercuric [Hg^2+^/Hg(II)] and mercurous [Hg^+^/Hg(I)] forms [218]. It can also occur as organic (or organomercuric) forms, such as the methylmercury (MeHg) ion (HgCH_3_^+^) and its compounds methylmercury chloride (CH_3_HgCl), methylmercury hydroxide (CH_3_HgOH), dimethylmercury and phenylmercury, identified as the most toxic forms of Hg [219,220]. The occurrence of these different chemical species depends on the environmental physicochemical features and how they are metabolized by different biological processes that occur in the local microbiota [217]. While Hg^0^ occurs mainly in the atmosphere, mercuric species [Hg(II)] are dominant in water, soil, and sediments and methylmercury (MeHg) in biota [221].

Mercury is a natural component of the Earth’s crust, often found as salts such as mercury sulfide (HgS, known as cinnabar) and other sulfate minerals (e.g., HgSO_4_), mercury oxide (HgO), mercury chloride (HgCl_2_), or as elemental mercury [222]. It can be released into the atmosphere through natural events such as volcanic activity, geothermal sources, biomass burning, and soil–water–air exchanges [223]. Both biotic (including bacteria) and abiotic (e.g., meteorological conditions and human activity) processes are involved in the transformation of mercury (geochemistry mercury cycle) into different inorganic and organic forms, as well as the gaseous element that returns to the atmosphere and contributes to its wide dispersion [224]. Nonetheless, 75% of the global mercury input and distribution to the environment is caused by extensive anthropogenic use [225], making it one of the most prevalent and persistent environmental pollutants [215].

Historical records reveal the use of quicksilver (liquid metallic mercury) in ancient Greek, Indian, Persian, Arabic, and Chinese medicine and alchemy [226,227]. In fact, it has been employed in traditional Chinese medicine for over 3000 years [226]. Additionally, evidence suggests that this metal was used as a preservative in Egyptian tombs [226]. Mercury compounds gained significant importance in medical applications during the late 15th century in Europe, particularly in the treatment of syphilis [228]. Moreover, the use of mercury became common in the 20th century in many applications (e.g., dental amalgam fillings; drug preservatives; antiseptics) [217,229,230]. Currently, it is still used in very small amounts as a preservative in some human and animal vaccines and pharmaceuticals, in the form of ethylmercury (known as thiomersal) [59]. In the agri-food sector, mercury was also used for decades, until the mid/late 20th century, in pesticides, mainly insecticides and fungicides, in the form of mercurous chloride and ethylmercury [230,231,232]. Although mercury contamination from industrial sources has declined globally in recent years due to stricter regulations (mainly due to the Minamata Convention on Mercury involving several countries worldwide) [232,233], anthropogenic processes are still responsible for a significant input of mercury into the environment [221,233]. Among the main activities that have been contributing to environmental contamination with mercury are cinnabar mining, coal combustion for energy production (an important source of atmospheric mercury), cement production, metal processing (gold, silver), waste incineration (from urban, medical, and industrial sources), chloralkali and steel industry, and the production of electric equipment, paints, and wood [223,232,234].

The extensive use of mercury in different applications has led to severe pollution in aquatic and terrestrial ecosystems. In recent years, a wide range of mercury concentrations have been found in soil (topsoil/agricultural land: 0–8889 mg/kg), water (marine sediments: 0.0023–5330 mg/kg; marine water: 0.5–27,060 ng/L; surface freshwater: 1.6–28.7 ng/L) [175,235,236,237,238], and across food webs, particularly in aquatic ecosystems where predatory fish (e.g., dusky grouper, barracuda, and porbeagle) bioaccumulate mercury (sea fish: 0.001–3.1 mg/kg; estuarine/freshwater fish: 0.04–1.74 mg/kg) [238,239]. Given the wide distribution of mercury in the environment and the abundance of bacteria on Earth, microorganisms are commonly exposed to and affected by toxic levels of mercury [240]. As a result, there is a widespread prevalence of the genetic determinants of mercury tolerance among bacterial populations, which allows their survival and adaptation in the presence of this toxic element in diverse environments. However, the mechanisms underlying mercury toxicity in bacterial cells are still not fully understood and continue to be the subject of study. Mercury exhibits a similar chemical reactivity to other metals (e.g., cadmium, lead, and arsenic) within cells, where it binds to sulfhydryl groups of enzymes and proteins [241], causing changes in the protein structure and often loss of function [242]. Recently, the affinity of mercury for the low molecular weight thiol molecules cysteine and glutathione (the most prevalent) and for proteins was described as involved in the replacement of essential zinc cofactors in DNA-binding proteins, which are involved in the transcription of tRNA genes and DNA repair, vital for many cellular functions [240].

**Figure 6:**
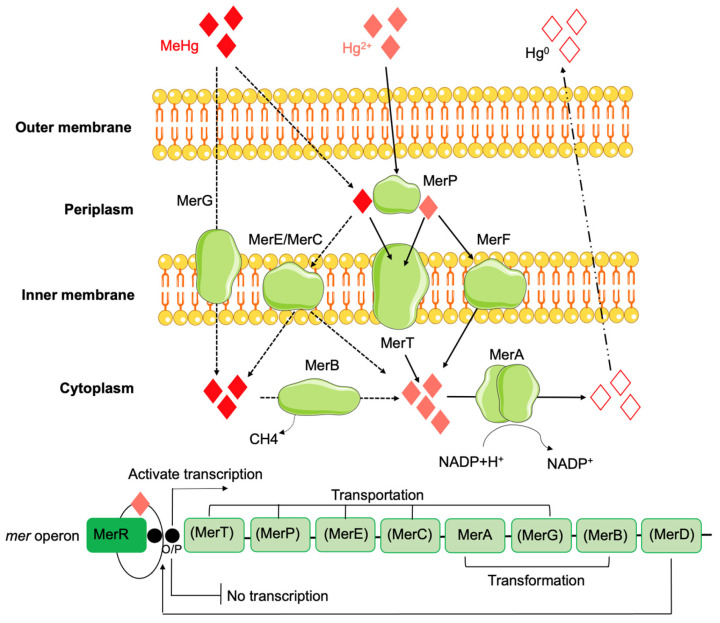
Generic model of the bacterial *mer* operon system. The *mer* operon genes are indicated below the illustration, with those in parentheses representing genes with variable presence in *mer* operons. Despite the variability of *mer* determinants in both Gram-positive and Gram-negative bacteria, overall *mer* expression is regulated by the MerR protein. The red diamonds represent the different types of Hg (MeHg—methylated Hg, Hg^2+^—inorganic Hg, Hg0—elemental Hg) (Adapted from [215]). The figure was partly generated using Servier Medical Art, provided by Servier, licensed under a Creative Commons Attribution 3.0 unported license.

Bacterial tolerance to mercury has been described in various Gram-positive and Gram-negative species from diverse sources (e.g., natural environments such as water, soil, and glaciers) or in human commensal/pathogenic bacteria [243,244,245,246] but is mainly associated with environments contaminated by mercury [247]. In fact, the first description of bacterial mercury tolerance (phenotypic feature) occurred at a time when mercurial compounds were widely used as topical disinfectants and antiseptics in hospitals, communities, and food-producing animals [248,249], and it was observed in a clinical isolate of *S. aureus* also resistant to penicillin [250]. At the same time the role of some anaerobic bacteria in the geochemistry of mercury, participating in the production of the most toxic form, methylmercury, was recognized in aquatic bottom sediments and fish [251]. To cope with mercury toxicity, bacteria have evolved the ability to convert toxic forms of mercury into nontoxic or relatively less harmful species, including the reduction of the highly reactive Hg^2+^ to metallic Hg^0^ (relatively inert, water-insoluble, and volatile) [252,253] or the degradation of organomercury compounds to inorganic mercury [248]. The *mer* operon is the most extensively studied cluster of genes that leads to mercury tolerance. It is highly variable among bacteria [248,254] and allows them to resist both inorganic and organic forms of mercury, known as narrow- or broad-spectrum mercury tolerance operons, respectively [215]. They typically consist of a combination of operators, regulators, promoter genes, and functional genes (e.g., *merT*, *merP*, *merE*, *merC*, *merA*, *merG*, *merB,* and *merD*), all or part of which are present, which contribute to the proper functioning of the operon system [247] (Figure 6).

The central enzyme in the mercury detoxification system is the mercuric reductase—MerA (encoded by the *merA* gene) [252], a cytosolic flavin disulfide oxidoreductase, which uses NAD(P)H as a reducing agent [248]. This protein is responsible for the volatilization of mercury, catalyzing the conversion of Hg^2+^ to Hg^0^ [255], and it is present both in narrow- and broad-spectrum *mer* operons [215]. While exhibiting a similar function role, variations in MerA amino acid sequences have been observed among Gram-positive and Gram-negative bacteria [255], suggesting a distinct ancestral origin of the *mer* operon for these two bacterial groups during the course of evolution [255]. In addition to MerA, a cytoplasmatic organomercury lyase—MerB (encoded by the *merB* gene) might also occur, allowing bacteria to resist organomercurials [215], catalyzing the demethylation of organic mercury compounds by lysing the carbon-Hg bond, transforming it into relatively less toxic Hg^2+^, which is then reduced by MerA to form Hg^0^ [215]. Therefore, the *merB* gene is associated only with the broad-spectrum *mer* operon [215]. The presence of the *merB* gene is more common in Gram-negative *mer* than in Gram-positive operons [248].

Other functional genes are primarily related to mercury transport and may include: *merT*, encoding an inner cytoplasmic membrane (MerT) protein responsible for accepting organic and inorganic mercury from MerP and transporting it to the cytoplasmic side of the membrane [248]; *merP*, which encodes a periplasmic scavenger protein that aids in the exchange of Hg^2+^ in the early transmembrane domain of MerP to MerT [215,248]; *merE*, which encodes a transport protein (MerE) that helps transport both inorganic and organic mercury compounds across the bacterial cell membrane into the cytoplasm [215,248]; and *merC*, which encodes an inner membrane-spanning transporter protein (MerC), which helps transport inorganic (Hg^2+^) and organic (C_6_H_5_Hg) mercury from the periplasm to the cytosol [215]. Additionally, *merG* is responsible for decreasing the cell membrane’s permeability to phenylmercury (since it and other organomercurials can potentially undergo simple diffusion [248]), contributing along with *merB* to broad-spectrum resistance against mercurial compounds [256]. The *merR* gene is associated with mercury tolerance expression, as it encodes an Hg^2+^-dependent transacting activator–repressor protein (MerR), which activates the *mer* genes in the presence of Hg^2+^ or represses it when a deficiency in Hg^2+^ occurs [257]. Other genes are also involved in the regulation of the *mer* operon, including the *merD* gene, which encodes a regulatory protein (MerD), responsible for the downregulation of the mercury tolerance system [215], and the *merO* gene, which is the operator region linked to the *merR* gene, responsible for upregulating and downregulating the expression of the *mer* operon genes [215]. A mercury tolerance phenotype associated with the presence of only the *merR* and *merA* genes was recently described in *Enterococcus* spp., with MICs to HgCl_2_ ranging between 16 and 64 μM, contrasting with those of 4–8 μM among isolates without such genes [150].

Mercury tolerance determinants are often located on the chromosome or plasmids of Gram-positive and Gram-negative bacteria, usually as components of transposable elements, in a striking diversity of arrangements [248]. The *mer* operon was first described in Gram-negative bacteria associated with Tn*501* and related transposons [246], and since then, several associations with plasmids and transposons have been identified in bacteria from natural environments [258] or with clinical relevance, including pathogenic strains of *E. coli* (e.g., genomic island GI-3) [259] and *S*. Typhimurium (e.g., GI-DT12 containing a Tn*21*-like transposon) [260]. In Gram-positive bacteria, *mer* operons have been found in diverse MGEs, including in *S. aureus* [e.g., plasmid pTW20_1 borne SCCmec (beta-lactamase) cassette] [261] and in *E. faecalis* and *E. faecium* (e.g., chromosomal Tn*5385*-like, pPPM1000) isolated from human (clinical) and animal samples, respectively [253,262]. The same mercury tolerance-associated transposons or plasmids often carry ARGs, which makes them potential vectors of multiple genes involved in co-resistance and co-selection events. Shortly after the first description of mercury tolerance in *S. aureus* resistant to penicillin, a plasmid (pI285) carrying both mercury tolerance and penicillin resistance genes was identified [189,263], along with other metal tolerance genes (arsenic/antimony, lead/zinc, and cadmium) [189]. In recent years, several reports have been published on the co-occurrence of mercury tolerance, ARGs, and biocide tolerance genes in the same MGEs, including in conjugative plasmids [253,264,265,266]. Specific associations of mercury tolerance genes with aminoglycosides [e.g., *aac(3)-IV*, *aadA*], sulfonamides (e.g., *sul*), or tetracycline [e.g., *tet(A)*] were described in the plasmids of *Klebsiella*, *Escherichia*, *Salmonella*, and *Enterobacter* isolated from diverse sources (human, animal, wastewater, and sludge) [133,134,267]. Additionally, the co-location of *mer* operon genes with β-lactams genes (*bla*_CTX-M_, *bla*_OXA_, or *bla*_TEM_) has also been described in the plasmids of *K. pneumoniae*, *E. coli,* and *Salmonella* from clinical, surveillance, food, and environmental samples [133,134,268,269,270]. In Gram-positive bacteria, particularly *Enterococcus* spp. from different sources (e.g., animal, healthy human, clinical, and hospital sewage), mercury tolerance genes have been co-located on plasmids with ARGs, mainly for erythromycin [*erm(B)*], tetracycline [*tet(M)*, *tet(L)*], aminoglycosides [*aadE*, *aadK*, *aac(6′)-aph(2′)*], and vancomycin (*vanA*) [150,253]. The distribution of mercury tolerance genes in MGEs along with ARGs genes highlights the potential impact of mercury on the co-transfer and dissemination of such determinants among bacteria of different sources.

## 3. Organic Acids

Organic acids are organic compounds with acidic properties [271], widely distributed in nature, either as natural constituents of plants and animals or metabolites of the activity of microorganisms (e.g., microbial fermentation) [55,272,273]. The most common organic acids comprise carboxylic acids, distinguished from other acids by the presence of the –COOH functional group, to which an organic group or a hydrogen atom may be attached [274]. Among this group of compounds are the straight chain saturated monocarboxylic acids and their derivates such as unsaturated (e.g., cinnamic and sorbic), hydroxylic (e.g., citric and lactic), phenolic (e.g., benzoic, cinnamic, and salicylic), and multi-carboxylic (e.g., azelaic, citric, and succinic) acids [274]. Chemically, organic acids are classified based on the number of hydroxy or carboxy functional groups and double bonds of carbon–carbon in their structures [271,275]. Other features, such as the nature of the carbon chain (aromatic, aliphatic, alicyclic, and heterocyclic) and saturation properties are important to categorize these compounds [275]. The number of carboxyl groups or other functional groups (e.g., alcohol, phenol, thiol, enol, and OSO_3_H) determines the compounds’ acidity [271]. In general, organic acids are weak acids not dissociating completely in the presence of water [271].

Organic acids are suspected to have been used in their natural form since prehistoric times [272,276], having a long tradition in the preservation of food products [277]. Acting mainly in the inhibition of microbial growth, these compounds prevent the deterioration of food products and extend their shelf life, especially the most perishable ones [278,279]. Originally, they began to be used as fungistats in animal feed [279], and with the discovery of their potential microbiocidal activity, they soon became widely applied in many products [279]. Currently, several organic acids and their salts are listed as food and feed additives in European legislation, most acting as preservatives and acidifiers (e.g., acetic, citric, formic, malic, fumaric, lactic, propionic, phosphoric, and sorbic) [280,281]. In food-producing animals, organic acids have been suggested as alternatives to other antimicrobials for use in nonclinical animal management practices [55,279]. Thus, dietary supplementation with organic acids (e.g., fumaric, lactic, citric, formic, malic, sorbic, and tartaric) in the feed and drinking water of animals for food production has become a common practice, given the benefits associated with weight gain and feed efficiency improvement [55,282]. In particular, the use of blends of various acids or their salts has been shown to enhance the beneficial effects of organic acids, improving the feed conversion ratio [283,284]. Additionally, general recognition of the safety of organic acids in food products has led to their wider application as sanitizers, not just in the food production setting (e.g., disinfection of surfaces and equipment in food production settings, including slaughterhouses) but also in food products (e.g., disinfection of fruits and vegetables or animal carcasses) [285,286]. In Europe, the application of organic acid solutions (e.g., lactic, acetic, and peroxyacetic acids) to reduce the microbial surface contamination of animal carcasses and meat has been evaluated by the EFSA [287,288], and the use of lactic acid is currently authorized in bovine carcasses [71]. Also, the application of organic acids (e.g., citric acid and succinic acid) has been tested for plant protection against phytopathogens (as a bactericide, fungicide, and nematicide) [289], although only acetic acid is currently authorized as an herbicide by some EU countries [290]. In recent years, promising new approaches have been explored in the food industry, including the use of organic-acid-based antimicrobial packaging, which combined with different preservation technologies contributes to increasing the shelf life of products [278].

The effectiveness of organic acids as antimicrobial agents relies on their ability to penetrate cell membranes as protonated acids [291]. Organic acids show a great ability to penetrate the cell wall, which makes them compounds with higher antimicrobial activity than the highly dissociated inorganic acids at the same pH level [278]. This feature is related to the ability of the organic acid to exist in a pH-dependent equilibrium between the undissociated and dissociated state [292]. The undissociated form is predominant at low pH and is primarily responsible for antimicrobial activity as it can freely diffuse across the cell membrane into the cytoplasm [293]. Once inside the cell, the higher pH will promote acid dissociation, resulting in the release of charged anions and protons and their accumulation in the cytoplasm. This creates not only an intracellular pH shift out of the optimal range for enzyme activity, affecting protein and DNA/RNA synthesis [273,294,295,296], but also hinders the proton motive force affecting energy production and inhibits the cell’s ability to re-alkalinize its cytoplasm [297]. In fact, pH homeostasis is a critical factor for cell growth and metabolism, influencing nutrient uptake and utilization, substrate degradation, and protein and nucleic acid synthesis [273]. Other mechanisms involved in microbial inactivation might include the disruption of metabolic processes (through the increase of osmolarity) and membrane functions [273,276], along with the growth inhibition, where certain organic acids (e.g., fumaric) act as chelators, binding to micronutrients [271]. Since the undissociated form of the acid is responsible for the antimicrobial effect, the pKa dissociation constant is an important factor, representing the pH at which 50% of the acid is dissociated. Thus, the higher the pKa of an organic acid, the more effective it will be, a factor further potentiated by other variables, including increasing the carbon chain length and the degree of unsaturation of the acid, acid concentration, exposure time, or temperature [292,293]. The efficacy of the organic acid will also depend on the specific type of microorganism targeted [293].

In contrast to other acids, peracetic acid (also known as peroxyacetic acid—PAA), widely used in the food and healthcare industries [298], also acts as a strong oxidant [299]. This organic peroxide (a synthetic chemical) is available in the form of a quaternary equilibrium mixture containing acetic acid, hydrogen peroxide, PAA, and water [300]. Thus, PAA combines the active oxygen characteristics of a peroxide within an acetic acid molecule [301] with the PAA, showing the highest biocidal activity [302]. Although there are few descriptions of the PAA’s mode of action as an antimicrobial compound, its activity is assumed to be similar to other peroxides and oxygen agents [299,303], causing oxidative stress in the cell by oxidizing and disrupting the sulfhydryl and sulfur bonds in proteins, enzymes, and other metabolites [299,301]. It can also act on the lipoprotein cytoplasmic membrane, disrupting its chemiosmotic function [301]. Intracellular PAA can also oxidize essential enzymes and impair vital biochemical pathways, active transport across membranes, and intracellular solute levels [301]. The pH is one of the most important factors of PAA activity, affecting the acid–base balance of PAA, which in turn affects the generation of free radicals [300]. The pKa value of PAA is 8.2, which means that under acidic conditions the predominant species is the undissociated acid form. At acidic-neutral pH (3–7), reactive radicals (e.g., OH·) increase [300], which contributes to the oxidizing properties of PAA. Additionally, in acidic environments (pH < 5.5), the decomposition of PAA to acetic acid by protonation and the release of protons during this process [304] may also contribute to the antimicrobial activity of PAA.

Bacteria are often exposed to both strong and mild acidic environments, either within the human/animal host (e.g., dental plaque, gastrointestinal tract, and macrophage phagosome) or outside in other human-associated niches, such as food processing and preservation [305], which creates a major challenge for the cell in maintaining pH homeostasis. In general, neutralophilic bacteria can grow at external pH values between ~5.5–9.0, while maintaining a cytoplasmic pH between ~7.2–7.8 (data reported for *E. coli*) [306]. However, when exposed to acid stress (pH 2.5–3.0), neutralophilic bacteria have evolved multiple tolerance or resistance mechanisms, responsible for increasing bacterial survival [305]. Cytoplasmic pH is buffered by small molecules (e.g., amino acids, proteins, polyamines, polyphosphate, and inorganic phosphate), representing a passive system in regulating pH homeostasis [307]. However, to counteract acid stress, active systems involving physiological, metabolic, and proton-consuming mechanisms are essential [307]. Common mechanisms involved in bacterial acid tolerance and part of the active systems include the decarboxylation of amino acids (e.g., glutamate, arginine, or lysine), the F_1_-F_0_-ATPase proton pump, and alkali production [308].

**Figure 7:**
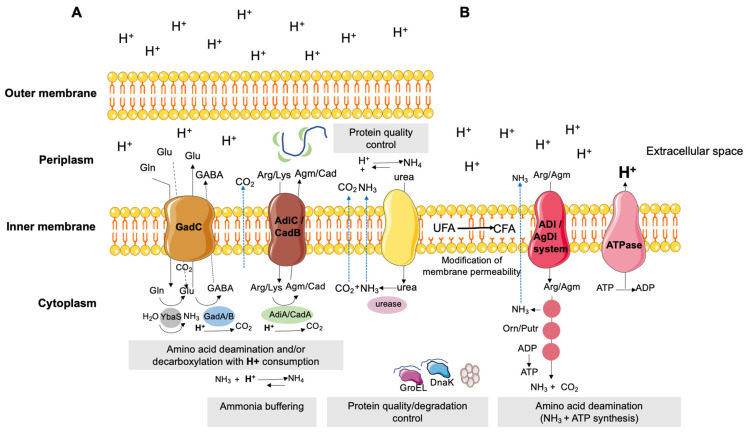
Bacterial mechanisms for responding to acidic pH stress. The image illustrates some of the best-studied acid response mechanisms used by Gram-negative (**A**) and Gram-positive (**B**) bacteria (adapted from [291]). Abbreviations: ADP—adenosine diphosphate, ATP—adenosine triphosphate, Arg—arginine, Agm—agmatine, Cad—cadaverine, GABA—Glu/γ-aminobutyrate acid, Gln—glutamine, Glu—glutamate, Lys—lysine, Orn—ornithine, Putr—putrescine. The figure was partly generated using Servier Medical Art, provided by Servier, licensed under a Creative Commons Attribution 3.0 unported license.

The decarboxylation of amino acids is an enzyme-catalyzed reaction that consumes protons [291]. Often called amino acid-dependent acid resistance systems, four distinct systems may be involved in the bacterial defense against acid damage: (a) the glutamic-acid-dependent acid resistance (GDAR) system; (b) the arginine-dependent acid resistance (ADAR) system; (c) the lysine-dependent acid resistance (LDAR) system; and (d) the ornithine-dependent acid resistance (ODAR) system [307]. The GDAR system is present in several bacteria such as *E. coli*, *Shigella flexnerii*, *L. monocytogenes*, *Lactobacillus reuteri,* and *Enterococcus avium* [308,309] and provides robust protection against extreme acid stress [310,311]. This system is responsible for catalyzing the conversion of protonated glutamate (Glu) to Glu/γ-aminobutyrate acid (GABA) and carbon dioxide, followed by the export of GABA through the GadC antiporter in exchange for a new extracellular Glu molecule (Figure 7) [308]. Recently, the *gad* gene (glutamate decarboxylase) was described in isolates of *E. coli* from chicken meat [43], suggesting an important feature for bacterial survival in food-producing animal environments, particularly poultry, where acidic pH can occur in different contexts (e.g., feed with organic acids additives, the gastrointestinal tract of animals, and processing plants using acidic disinfectants). In *Salmonella enterica*, the presence of genes associated with the ADAR (*adiA*—arginine decarboxylase and *adiC*—arginine–agmatine antiporter) and LDAR (*cadA*—lysine decarboxylase and *cadB*—lysine–cadaverine antiporter) systems has also been described as an important feature for neutralizing and surviving acid stress [312], allowing bacterial survival in harsh acidic environments (e.g., stomach and phagolysosomes), determinant for the dissemination capacity and virulence of this food-borne pathogen. Additional decarboxylation pathways have been less studied in other bacteria, including tyrosine decarboxylation associated with the acid response mechanism in several lactic acid bacteria, such as *Enterococcus* spp., giving them a competitive advantage in acidic environments [313].

Deamination of amine-containing amino acids [e.g., arginine (Arg), agmatine (Agm), or glutamine (Gln)] and the urease system are also important acid response mechanisms, associated with the production of basic compounds such as ammonia (NH_3_), important to avoid a critical drop in internal pH (Figure 7) [291]. In the urease system, urea is hydrolyzed to NH_3_ and carbon dioxide (CO_2_) by ureases [308]. Furthermore, the conversion of Gln to Glu by acid-activated glutaminase (YbaS), of Arg to ornithine (Orn) by arginine deaminase (ADI system), and of Agm to putrescine (Putr) by agmatine deiminase (AgDI system) releases NH_3_ and CO_2_ (Figure 7) [305,308]. NH_3_ directly neutralizes protons and regulates the cytoplasmic pH [314].

Another important mechanism relies on the activity of proton pumps (e.g., H^+^-ATPase, symporter, and antiporter) that promotes proton efflux in a proton motive force (PMF) dependence system [273]. The efflux of protons out of the cell is an ATP-consuming process (Figure 7), which leads to a depletion in the energy available to cells and, consequently, affects their survival [273]. The F_1_-F_0_-ATPase is a bifunctional proton pump, that catalyzes the synthesis and hydrolysis of ATP [315]. This multi-subunit enzyme uses the energy released from the movement of protons across cell membranes to generate ATP and, in a reverse reaction, hydrolyzes ATP to export protons across the membrane, thereby maintaining pH homeostasis particularly in acidic environments [308]. In fact, induction of the F_1_-F_0_ operon by exposure to acidic pH suggests that this enzyme plays a critical role in acid resistance in several bacteria [308].

In contrast to inorganic acids (e.g., hydrochloric acid), which primarily lower cytoplasmatic pH, organic acids have the additional ability to accumulate as intracellular anions [312]. When these anions accumulate in high concentrations within bacterial cells, they can exert inhibitory effects. As a result, bacteria have evolved mechanisms to efflux these anions using membrane pumps [316]. Consequently, the mechanisms involved in the acid stress response that are induced by organic acids appear to differ from those triggered by inorganic acids [317]. However, it is important to note that cells adapted to withstand inorganic acids also acquire resistance to acid stress induced by organic acids and vice versa [312]. Some organic acid tolerance mechanisms have been explored, mainly in organic acid-producing bacteria (e.g., *Acetobacter*, *Lactobacillus*) [318,319]. In addition to those previously described (e.g., amino acid decarboxylation, proton pumps, and neutralization processes), additional mechanisms have been reported, for example, the PQQ-ADH (pyrroloquinoline quinine-dependent alcohol dehydrogenase) system, known to be involved in tolerance to acetic acid in acetic acid bacteria [320]. Interestingly, in acetic acid bacteria, the GDAR acid resistance system is absent, and the urea degradation is down-regulated after acetic acid production [317]. In the case of PAA, certain bacteria, including pathogenic strains such as *S. enterica* [312], can induce the expression of genes associated with oxidative stress (e.g., SoxRS, OxyR, and PerR regulon), with such induction being associated with a protective response against the activity of PAA [321]. In fact, in-use concentrations of PAA for food and feed area disinfection (20–3000 mg/L for Product Type-PT4) have recently been described as being, in some cases, lower than the MIC (60–70 mg/L) and MBC (70–90 mg/L) shown by poultry associated *S*. *enterica* strains [322].

Unlike metals, limited information on the co-selection of antibiotic resistance and acid tolerance is available. A recent study using metagenomic approaches revealed the co-occurrence of the *pmrA/B/C* polymyxin resistance genes and *actP* acid resistance gene [323]. Furthermore, other acid resistance genes (e.g., *gadE*, *hdeA*, *mdtE*, *mdtF*, *gadW*, *gadX*, and *gadA*) were co-located with metal tolerance genes (mainly arsenic—*arsA/B/R*) in the same contig [323]. In the case of PAA, the literature suggests the absence of a strong association between tolerance to PAA and resistance to antibiotics. For example, some studies have shown that exposure of *S. enterica* strains to subinhibitory concentrations of PAA (MIC/2; ~0.040 mg/mL) [324] resulted in increased resistance to streptomycin and neomycin [325], but this association appears to be strain-specific [325]. On the other hand, a study involving more than 500 *S. enterica* isolates from Danish pig slaughterhouses found little evidence of an association between increased MIC for PAA and antibiotic resistance [326]. Likewise, *E. faecium* exposed to low doses of PAA did not show changes in the abundance of ARGs [327]. In addition, no antibiotic cross-resistance was observed in *L. monocytogenes* from food production plants exposed to PAA [328]. Indeed, according to the EFSA, there is no evidence to suggest that PAA can lead to acquired antibiotic resistance [329]. However, a recent study has suggested that reactive oxygen species may promote antibiotic resistance by increasing the expression of the MDR efflux pump via activation of the SoxRS redox regulon [330], a mechanism that should not be ruled out due to the oxidative stress created by PAA.

## 4. Conclusions

While antibiotic misuse and overuse remains the main driving force behind the emergence of AMR in the agri-food sector, there is growing recognition of the potential role of other antimicrobial compounds in this problem. Metals, including copper commonly found in feed, as well as pollutants such as arsenic and mercury that enter the food chain, can potentially contribute to the co-selection of ARB, often sharing metal tolerance genes and ARGs in diverse mobile genetic contexts. However, for organic acids, only a limited number of studies exploring the potential link between bacterial tolerance to these compounds and the emergence of AMR are available. Moreover, such studies provide limited data on the impact of organic acids on the selection of particular MDR strains (e.g., serotypes, clones, and antibiotic resistance profiles) as they only include a small number of isolates with non-characterized epidemiological and genetic backgrounds. More data on the molecular mechanisms explaining adaptive features to different organic acids are also still needed for different bacteria.

To effectively address the challenge of AMR in the food chain, future research should provide better and larger data on the epidemiological and genomic backgrounds of bacteria tolerant to metals or organic acids, as well as on the characterization of the genetic contexts accumulating and mobilizing tolerance genes to these stresses. It is also critical to assess the minimum selective concentrations of metals and organic acids for particular MDR bacterial clones, serotypes, and MGE in food contexts worldwide with diverse selective pressures and to investigate the ecological factors promoting the horizontal gene transfer events of AMR or ARG expression. Additionally, understanding the long-term impact of continued metal and organic acid exposure on AMR dynamics, especially in bacteria of clinical relevance to humans and animals, is essential. Employing advanced genomic and metagenomic techniques in longitudinal studies will help unravel the genetic basis and shifts of bacterial communities’ tolerance under the same stresses and after different controlling interventions, whose data are currently limited. Thus, future approaches to the problem will need to adopt a One Health strategy to examine the interconnectedness of metal and organic acid tolerance and AMR across humans, animals, and the environment. This will provide comprehensive insights, allowing the optimization of the use of metals and organic acids to mitigate the microbial risks associated with food production and to prevent pollution. It will enable more effective management practices, ensuring the long-term effectiveness of antimicrobial treatments for all.

## Data Availability

Not applicable.
